# Established and emerging techniques for the study of microglia: visualization, depletion, and fate mapping

**DOI:** 10.3389/fncel.2024.1317125

**Published:** 2024-02-15

**Authors:** Bianca Caroline Bobotis, Torin Halvorson, Micaël Carrier, Marie-Ève Tremblay

**Affiliations:** ^1^Division of Medical Sciences, University of Victoria, Victoria, BC, Canada; ^2^Centre for Advanced Materials and Related Technology, Victoria, BC, Canada; ^3^Department of Medicine, University of British Columbia, Vancouver, BC, Canada; ^4^Department of Surgery, University of British Columbia, Vancouver, BC, Canada; ^5^British Columbia Children’s Hospital Research Institute, Vancouver, BC, Canada; ^6^Département de Psychiatrie et de Neurosciences, Faculté de Médecine, Université Laval, Québec City, QC, Canada; ^7^Axe neurosciences, Centre de Recherche du CHU de Québec, Université Laval, Québec City, QC, Canada; ^8^Department of Molecular Medicine, Université Laval, Québec City, QC, Canada; ^9^Department of Biochemistry and Molecular Biology, University of British Columbia, Vancouver, BC, Canada; ^10^Department of Neurology and Neurosurgery, McGill University, Montréal, QC, Canada

**Keywords:** microglia, microglial markers, fate mapping, electron microscopy, positron emission tomography, reporter genes, microglial depletion, Cre/lox systems

## Abstract

The central nervous system (CNS) is an essential hub for neuronal communication. As a major component of the CNS, glial cells are vital in the maintenance and regulation of neuronal network dynamics. Research on microglia, the resident innate immune cells of the CNS, has advanced considerably in recent years, and our understanding of their diverse functions continues to grow. Microglia play critical roles in the formation and regulation of neuronal synapses, myelination, responses to injury, neurogenesis, inflammation, and many other physiological processes. In parallel with advances in microglial biology, cutting-edge techniques for the characterization of microglial properties have emerged with increasing depth and precision. Labeling tools and reporter models are important for the study of microglial morphology, ultrastructure, and dynamics, but also for microglial isolation, which is required to glean key phenotypic information through single-cell transcriptomics and other emerging approaches. Strategies for selective microglial depletion and modulation can provide novel insights into microglia-targeted treatment strategies in models of neuropsychiatric and neurodegenerative conditions, cancer, and autoimmunity. Finally, fate mapping has emerged as an important tool to answer fundamental questions about microglial biology, including their origin, migration, and proliferation throughout the lifetime of an organism. This review aims to provide a comprehensive discussion of these established and emerging techniques, with applications to the study of microglia in development, homeostasis, and CNS pathologies.

## Introduction

**FIGURE 1:**
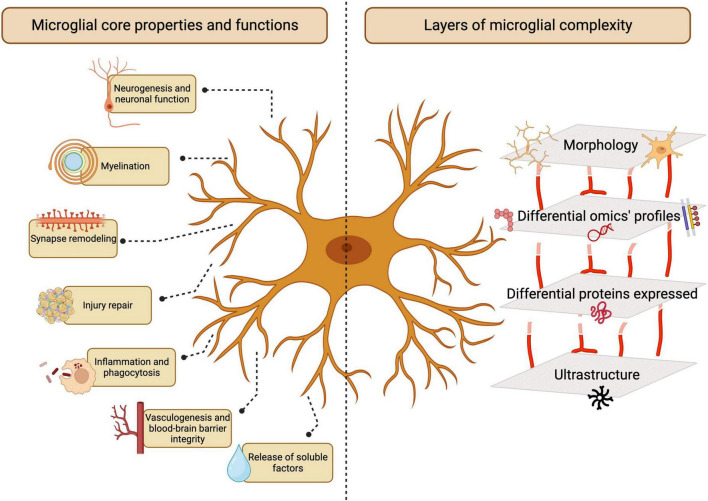
Microglia functions in the central nervous system (CNS). Microglia play essential and multifaceted roles in the CNS throughout homeostasis and disease. The study of microglia involves several layers of complexity, ranging from morphological analysis to emerging multi-omics-based characterization. Created with BioRender.com.

Microglia are the resident innate immune cells of the central nervous system (CNS). Originating in the yolk sac and colonizing the CNS during early embryonic development (Ginhoux et al., 2010; Gomez Perdiguero et al., 2015), microglia perform various physiological roles in the development and maintenance of CNS homeostasis, continuously monitoring their microenvironment and responding to cues (Šišková and Tremblay, 2013; Ransohoff and El Khoury, 2015; Augusto-Oliveira et al., 2019). During homeostasis, these cells notably assist in maintaining neuronal and glial populations, clear extracellular debris, and regulate axonal myelination, neuronal activity and neurotransmitter levels as pivotal players of CNS function, plasticity and integrity (Gomez-Nicola and Perry, 2015; Cornell et al., 2022). Dysregulation of microglial activities is linked to several neurodevelopmental, neurological and psychiatric disorders, leading to neuronal damage and cognitive impairment (Figure 1; Tremblay and Majewska, 2011; Kwon and Koh, 2020).

The wide range of microglial functions in the CNS is attributable to the established concept of microglia as a heterogeneous and dynamic cell population. Microglia coexist in a plethora of structurally and functionally distinct states, including homeostatic and disease-associated phenotypes (Keren-Shaul et al., 2017; Butovsky and Weiner, 2018; Deczkowska et al., 2018; Hammond et al., 2019; Li et al., 2019a; Stratoulias et al., 2019). These states are not fixed; rather, microglia undergo phenotypic shifts, often rapidly, in response to diverse stimuli (Nimmerjahn et al., 2005; Bennett et al., 2016; Krasemann et al., 2017). The microglial sensome, composed of various receptors and signaling molecules, enables these cells to detect changes in their environment and respond accordingly, actively regulating CNS homeostasis (Nimmerjahn et al., 2005; Hanisch and Kettenmann, 2007; Hickman et al., 2013; Rubino et al., 2018; Carrier et al., 2022). Understanding microglia is a challenging endeavor due to the complex nature of these cells, which express diverse receptors and exhibit different states/phenotypes that are context-dependent. These states are influenced by various factors such as age, environmental exposures, disease, and sex (Carrier et al., 2022; Bobotis B. C. et al., 2023; González Ibáñez et al., 2023; Picard et al., 2023). Moreover, multimodal approaches are often employed in order to distinguish these cells from non-parenchymal cells in the CNS and infiltrating myeloid cells. Through the combination of genetic labeling, imaging, and knockout (KO) studies with proteomic, metabolomic and transcriptomic analyses, our understanding of microglial behavior, phenotype, and function in the CNS has greatly advanced in recent years.

The field of immunology has advanced substantially through the use of imaging tools. From Élie Metchnikoff’s pioneering visualization of phagocytosis in 1882 (Tan and Dee, 2009) to modern techniques such as 3-dimensional electron microscopy (EM) and two-photon *in vivo* microscopy (Maco et al., 2014), the range of tools available for studying the CNS immune system has significantly expanded. Notably, this has enabled a more comprehensive understanding of the microglial sensome and the multi-dimensional mechanisms underlying microglial physiological and immunological functions (Hickman et al., 2013; Sierra et al., 2019; Carrier et al., 2021). As we strive to understand the role of microglia in both pathology and homeostasis, the ability to visualize, selectively modulate or deplete these cells across different contexts has become increasingly important (Hickman et al., 2013; Konishi and Kiyama, 2018). Characterizing the microglial sensome and identifying specific microglial markers are crucial endeavors to furthering understanding of the mechanisms underlying microglia-mediated CNS inflammation and microglial involvement in models of various disorders of the CNS (Hickman et al., 2013; Carrier et al., 2022). This review will examine emerging techniques for microglial imaging and visualization, through antibody-mediated labeling, EM, reporter models, and fate mapping, as well as strategies for the specific depletion or genetic modification of microglia to gain insights into their function. Given that microglia reside in the CNS, much of our knowledge of the roles of microglia in living organisms derives from work in rodents. Thus, we primarily focus on the study of microglia in mouse models, corroborating results with studies of human microglia where possible.

## Antibody-mediated identification of microglial markers

**FIGURE 2:**
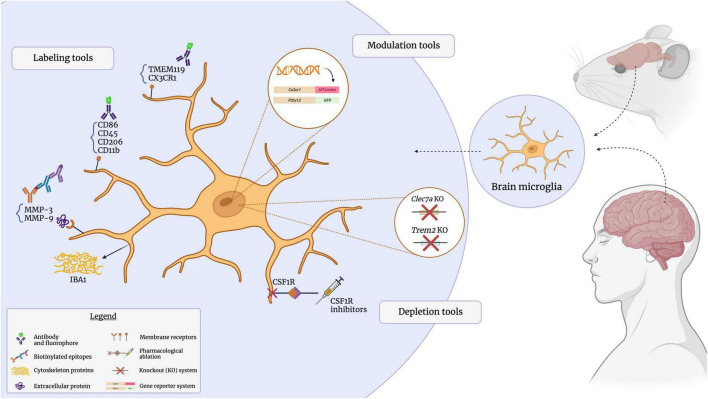
Visualizing the microglial sensome. The microglial sensome comprises a plethora of extracellular, intracellular, and secreted proteins. Antibody-mediated detection, as well as genetic and pharmacological approaches to selectively visualize and modulate these targets through immunostaining assays, reporter-based strategies, and knockout models, have greatly contributed to our understanding of microglial function. Created with BioRender.com.

Antibodies are versatile tools that can be used to identify, isolate, quantify, localize and visualize specific targets in a biological sample (Goldman, 2000). Researchers have adopted the use of antibodies for a wide range of specialized techniques, such as immunohistochemistry (IHC), enzyme-linked immunosorbent assays (ELISA), Western blotting, flow cytometry and fluorescence-activated cell sorting (FACS). Several commonly targeted microglial surface proteins, discussed in detail below, enable the antibody-mediated visualization of microglia in the CNS, since these cells express a great variety of receptors to mediate extensive interactions with other cell types and their microenvironment (Casali and Reed-Geaghan, 2021). The subcellular location of microglial proteins is an important consideration, as intracellular and extracellular protein targets require distinct labeling approaches and provide different insights into microglial populations and their dynamics (Figure 2). For instance, while membrane and extracellular proteins can shed light on microglial reactivity, inflammatory processes and interactions with other cell types (Szepesi et al., 2018; Jurga et al., 2020); intracellular proteins can elucidate cytoskeletal reorganization processes, with implications for cell migration and morphology (Sasaki et al., 2001; Ohsawa et al., 2004; Rosito et al., 2023). Microglia produce a number of cytokines and extracellularly-active enzymes (Kingham and Pocock, 2001; Lee et al., 2010; Smith et al., 2012). The cytokines and secreted proteins they release serve as distinctive markers for identifying microglia and offer valuable insights into their functionality, state, and phenotype. Specific microglial states are characterized by distinct profiles of released cytokines and differentially expressed genes; for instance, the disease-associated microglia (DAM; further discussed in the following section) downregulate homeostatic genes and reactive states of microglia are known to produce elevated levels of pro-inflammatory cytokines, such as IL-1, IL-6, and TNF-α (Smith et al., 2012; Šišková and Tremblay, 2013; Jurga et al., 2020; Paolicelli et al., 2022; Rosito et al., 2023). As certain microglial proteins are differentially expressed under inflammatory conditions, and can furthermore be induced on other cell types, validation of individual markers within the specific model of interest is advisable (Honarpisheh et al., 2020). Key microglial surface, intracellular and secreted proteins will also be summarized in Table 1.

**TABLE 1:** Summary of key microglial surface, intracellular and secreted proteins.

Protein	Location	Main function	Other cell types expressing the protein	References
CD11b	Membrane protein	Adhesion and inflammatory processes of the complement system	Monocytes, neutrophils, Natural Killer cells (NK), granulocytes, macrophages, dendritic cells (DCs)	Fischer and Reichmann, 2001; Greter et al., 2015; Martin et al., 2017; Agalave et al., 2020
CD86	Membrane protein	T cell activation	DCs, Langerhans cells, macrophages, and B cells	Hellström Erkenstam et al., 2016; Bai et al., 2017; Peng et al., 2019; Yusuying et al., 2022
CD45	Membrane protein	Protein tyrosine phosphatase, T-cell activation	Leukocyte common antigen	Martin et al., 2017; Sousa et al., 2018
CD68	Membrane protein	Innate inflammatory response, possible role in phagocytosis; regulation of antigen processing	Monocytic phagocytes, osteoclasts, Kupffer cells	Rice et al., 2017; Yeo et al., 2019; Swanson et al., 2023
CD206	Membrane protein	Endocytosis and phagocytosis	Astrocytes, macrophages, DCs, and endothelial cells	Hellström Erkenstam et al., 2016; Wu et al., 2021; Yusuying et al., 2022
TMEM119	Membrane protein	Proliferation, migration and genetic stability	DCs, fibroblasts, peritubular cells	Bennett et al., 2016; Satoh et al., 2016; Ibanez et al., 2019; Kaiser and Feng, 2019
P2RY12	Membrane protein	Detects ATP-derived particles; motility	Vascular smooth muscle cells, brown adipocytes, cholangiocyte primary cilia, osteoblasts, osteoclasts, DCs, lymphocytes.	Avignone et al., 2008; van Wageningen et al., 2019; Bisht et al., 2021
CX3CR1	Membrane protein	Microglia adhesion and migration; neural communication	Monocytes, macrophages, T helper cells, CD8+ effector/memory T cells, NK cells, γδ T cells, DCs	Liang et al., 2009; Tang et al., 2014; Pagani et al., 2015; González-Prieto et al., 2021
CLEC7a	Membrane protein	Glucan receptor; immune response via reactive oxygen species	Monocytes, macrophages, DCs, neutrophils, B cells	Bisht et al., 2016; Shi et al., 2020; Wang et al., 2022
TREM2	Membrane protein	Mediates transcription factors; synaptic pruning	Macrophages, DCs	Bisht et al., 2016; Krasemann et al., 2017; Filipello et al., 2018; Reifschneider et al., 2022; Gao et al., 2023
IBA1	Intracellular protein	Microglial cytoskeleton reorganization	Macrophages, monocytes, Hofbauer cells, Kupffer cells, Langerhans cells	Bisht et al., 2016; Ibanez et al., 2019; Shi et al., 2021
SALL1	Intracellular protein	Transcriptional regulator in homeostasis	Stem cells, oligodendrocytes, hepatocytes, astrocytes	Buttgereit et al., 2016; Salman et al., 2018; Scott et al., 2022
HEXB	Intracellular protein	Lysosomal processes, ganglioside degradation	Adipose progenitor cells, fibroblasts, thyroid glandular cells	Masuda et al., 2020; Sierksma et al., 2020; Jia et al., 2021
MMP-9 and MMP-3	Extracellular proteins	Cytokine activation in inflammatory processes	Neutrophils, macrophages, and fibroblasts	Woo et al., 2008; Lee et al., 2014; Kim J. et al., 2021

### Membrane-associated surface markers

Microglia present unique transcriptional signatures related to their activity and the environmental context. Under homeostatic conditions, certain surface proteins are highly expressed, such as the CX3-motif chemokine receptor (CX3CR1), the P2Y12 purinergic receptor (P2RY12), and transmembrane protein 119 (TMEM119), among others (Song and Colonna, 2018; Paolicelli et al., 2022). However, microglial states induced by pathological insults, such as the DAM and microglial neurodegenerative phenotype (MGnD), adopt a distinct gene signature characterized by downregulation of homeostatic *Cx3cr1*, *P2ry12*, and *Tmem119*, together with upregulation of *Trem2*, *Apoe*, *Clec7a*, and *Cd11c* (Keren-Shaul et al., 2017; Krasemann et al., 2017; Butovsky and Weiner, 2018; Deczkowska et al., 2018; Paolicelli et al., 2022). While their functions have not been fully elucidated, DAM are involved in phagocytosis, clearance of apoptotic cell bodies and inflammatory responses (Deczkowska et al., 2018; Dubbelaar et al., 2018; Casali and Reed-Geaghan, 2021). The case of DAM illustrates that certain markers can differentially identify key microglial states during homeostasis and pathology, and these will be the subject of the following discussion. These markers are often used in immunostaining but also are frequently employed in Cre/lox systems, knockout models, using FACS and other methods (Ruan et al., 2020; Perrone et al., 2023). As an illustration of the diversity of techniques used to study microglial markers, Li et al. (2019b) combined FACS, MACS, flow cytometry and q-PCR on whole adult mouse brain tissue to investigate differences between microglial and macrophage responses to intracerebral hemorrhage. Their findings revealed elevated expressions of Tmem119 and Sall1 in the sorted microglial cell population (Ly6g^–^CD11b^+^CD45*^int^*PI^–^), while Ccr2 and CD69 were highly expressed in the monocyte/macrophage sorted cell population (Ly6g^–^CD11b^+^CD45*^high^*PI^–^), highlighting the utility of multifaceted approaches using multiple markers to reliably distinguish microglia. Notably, microglia expressing both *Tmem119* and *Sall1* exhibited increased expression following intracerebral hemorrhage, whereas monocytes/macrophages maintained consistent levels of both Ccr2^+^/CD69^+^ in the sham and hemorrhage groups (Li et al., 2019b). As discussed in the following sections, distinct sets of markers are commonly used to identify microglia depending on which technique is employed.

CD11b, one of the first identified β-glucan receptors, is an abundant surface protein primarily expressed on cells of the myeloid lineage that has been reliably used for microglial visualization. CD11b assists in endothelial adhesion and regulates the uptake of complement-coated particles by phagocytosis (Xia et al., 1999; Nikodemova and Watters, 2012; Schmid et al., 2018). As CD11b is also expressed by peripherally-derived myeloid cells in the CNS (Fischer and Reichmann, 2001; Greter et al., 2015), it must be paired together with other markers to identify microglia more specifically. It is often co-utilized with universally expressed leukocyte surface proteins such as CD45, the combination of which has utility for identifying microglia by flow cytometry (Bruzelius et al., 2021).

CD45, present on all leukocytes in varying levels, and CD68, a lysosomal marker present in endosomes and lysosomes of the mononuclear phagocytic lineages [including microglia, border-associated macrophages (BAMs) and macrophages], can be used to identify microglia in the CNS and other immune cells (Hendrickx et al., 2017; Rangaraju et al., 2018; Waller et al., 2019; Bruzelius et al., 2021). CD45, a protein tyrosine phosphatase, plays a role in the proliferation and differentiation of immune cells (Penninger et al., 2001), but tends to be expressed at lower levels on microglia than on CNS-infiltrating myeloid cells during non-inflammatory conditions, while CD68 is associated with inflammatory responses, phagolysosomal activity, and the regulation of antigen presentation (Song et al., 2011; Chistiakov et al., 2017). Both CD45 and CD68 contribute to the microglial sensome under homeostatic conditions, although CD45^+^ and CD68^+^ signatures are associated with microglial reactivity in response to inflammation, trauma or pathology (Perego et al., 2011; Hoogland et al., 2015; Hendrickx et al., 2017; Rice et al., 2017; Swanson et al., 2023). In combination with other markers, such as ionized calcium-binding adaptor molecule 1 (IBA1) and CD11b, CD45 can aid in distinguishing microglia from other CNS infiltrated myeloid cells and non-parenchymal cell populations, using, for instance, flow cytometry (Waller et al., 2019; Bruzelius et al., 2021). Microglia are generally identifiable as CD11b^+^CD45*^int^*, in contrast to CD11b^+^CD45*^hi^*, being usually attributed to CNS-infiltrating myeloid cells or BAMs at a steady state (Greter et al., 2015; Martin et al., 2017; Rubino et al., 2018; Honarpisheh et al., 2020). Nevertheless, under specific conditions, microglia may exhibit CD45*^high^* expression. Studies conducted in models of stroke, cerebral amyloid angiopathy, and aging indicate that *Tmem119* and *P2RY12* expression is present in both CD45*^int^* and CD45*^high^* FACS-sorted cells myeloid cell populations (Honarpisheh et al., 2020). Moreover, a small population of CD11b^+^CD45*^high^* mononuclear phagocytes has been identified by flow cytometry in the surroundings of Aβ plaques in the 5XFAD mouse model of Alzheimer’s disease (AD) pathology, and these cells exhibit transcriptional similarities to DAM, such as upregulation of *Trem2* and *Cd11c* (Rangaraju et al., 2018). Additionally, CD44 is also often employed to emphasize CNS-infiltrated myeloid cells, given that macrophages, for example, typically exhibit CD44*^high^* expression. In pathology, microglia can exhibit CD44 expression, especially when exposed to lipopolysaccharide (LPS) and in degenerative states like amyotrophic lateral sclerosis. The findings also indicate that primary cultured microglial cells express CD44 upon exposure to LPS and interferon-γ. Moreover, the results align with increased CD44 expression observed following treatment with interferon-γ and TNF-α (Matsumoto et al., 2012; Liu S. et al., 2023).

Thus, the identification of microglia by flow cytometry in pathological conditions remains complicated, requiring fate mapping approaches to distinguish these lineages robustly. Additionally, distinguishing microglia from CNS-infiltrated macrophages is crucial for the precise characterization and understanding of the functionally of each cell type. Notably, BAMs form a distinct, specialized population naturally located in the choroid plexus (CP), meninges and perivascular spaces (Dermitzakis et al., 2023). Given the immune-privileged nature of the CNS, it is naturally constituted and protected by microglia, parenchymal tissue-resident macrophage, along with non-parenchymal cells, the BAMs. The resemblance to microglia is significant, as these cells are also derived from the yolk sac and share certain commonly expressed genes with microglia. For instance, they exhibit low expression levels of *P2y12* and *Tmem119*, while microglia express these genes at higher levels (Rajan et al., 2020). Single-cell approaches, using mouse enriched subdural meninges (SDM) CP*^epi^*-BAMs, have also demonstrated that BAM subsets express common DAM signature genes, including an upregulation of genes involved in lipid metabolism or phagocytosis, such as *Cst7*, *Apoe*, *Clec7a*, and *Lpl* (Van Hove et al., 2019). BAMs often express lymphatic-like gene signatures, such as *Pdpn* and *Lyve1*, frequently associated with cell migration. Expression levels naturally differ among distinct subpopulations of BAMs located in various non-parenchymal regions, such as the or meninges (Møllgård et al., 2023). Interestingly, microglia and BAMs share the same erythromyeloid progenitors (EMPs) in the yolk sac, however, fate mapping approaches revealed that these EMPs seem to depend on CD206 expression for their differentiation into microglia or BAMs (Masuda et al., 2022). While only a few microglia start to express CD206 at the embryonic day (E)12.5, BAMs start to highly express it at E10.5 (Masuda et al., 2022). BAMs will be further discussed in the subsequent fate mapping sections. To ensure the characterization and distinction of microglia, BAMs and peripherally derived myeloid cells, complementary techniques are often employed, such as flow cytometry, fate mapping and single-cell transcriptome analysis. IHC can also aid in discerning microglia from peripherally-derived myeloid cells, for example based on higher levels of IBA1 and TMEM119 expression in microglia (Kishimoto et al., 2019).

Both CD86 and CD206 characterize reactive microglia but are associated with distinct functional properties and microglial phenotypes, which are extensively examined in immunohistochemistry and flow cytometry. The C-type lectin CD206 plays a role in recognizing pathogens and clearing glycoproteins from the circulation (Suzuki et al., 2018; Tanaka et al., 2021). Not specific to microglia, CD86 and CD206 are also expressed by other cells in the CNS, such as astrocytes and BAMs, and throughout the body, being found in dendritic cells, macrophages/monocytes and B/T-lymphocytes (Xing et al., 2018; Sun et al., 2019; Trzupek et al., 2020). Upregulation of CD206 is observed in pathological conditions, such as glioma, where increased microglial pinocytosis and phagocytosis mediate the clearance of debris and resolution of inflammation (Tanaka et al., 2021). CD86, also a transmembrane protein, provides a costimulatory signal essential for the activation and proliferation of T cells. Hence it is abundantly expressed on antigen-presenting cells, including microglia, macrophages, dendritic cells, and B cells, and is upregulated in response to injury (Louveau et al., 2015; Hellström Erkenstam et al., 2016). Remarkably, it is seen an increase in both CD86^+^ and CD206^+^ subsets among brain CD11b^+^ cells, in models of traumatic brain injury, neonatal brain hypoxia-ischemia and cerebral ischemia-reperfusion (Jin et al., 2012; Hellström Erkenstam et al., 2016; Bai et al., 2017; Yusuying et al., 2022).

As microglia respond to many cues in the microenvironment, they also naturally express receptors associated with neuronal communication (Butler et al., 2021). CX3CR1 is widely expressed in the CNS, for instance, in macrophages and microglia, and its ligand, fractalkine (CX3CL1), is an important chemokine produced by neurons (Wolf et al., 2013; Szepesi et al., 2018). The CX3CL1-CX3CR1 axis mediates the development and plasticity of neuronal circuits, with functional consequences for brain connectivity, as notably shown by comparing the CX3CR1*^KO/KO^* mice with CX3CR1^*KO/*+^ mice (Drissen et al., 2019; Lauro et al., 2019). Under physiological conditions, CX3CR1-expressing microglia in the brain are extensively involved in dynamic surveillance and maintenance of brain homeostasis (Lauro et al., 2019). CX3CR1 signaling is also important for microglial motility and the regulation of the microglial response to injury and inflammation. Studies employing immunostaining and q-PCR in mouse models of AD pathology and ischemic stroke have shown that CX3CR1*^high^* microglia have increased migration to sites of pathology, whereas CX3CR1*^low^* microglia tend to remain in a surveillance state (Tang et al., 2014; Hickman et al., 2019). Similarly, CX3CR1-deficient microglia exhibit delayed migration toward sites of laser-induced injury and impaired repopulation following depletion (Liang et al., 2009; Zhang et al., 2018).

In recent years, TMEM119 had emerged as a microglial marker with high specificity (Bennett et al., 2016; Satoh et al., 2016). Initially identified as a regulator of osteoblast differentiation, TMEM119 is thought to regulate the Wnt/β-catenin pathway, important for proliferation, migration, and genetic stability, but further research is required to fully elucidate its functions in microglia (Satoh et al., 2016; Yang et al., 2021). Selectively expressed by yolk sac-derived cells in the CNS (e.g., microglia and BAMs in the CNS), TMEM119 broadly distinguishes resident microglia from infiltrating macrophages (Haynes et al., 2006; Segal and Giger, 2016; Bohnert et al., 2020; Vankriekelsvenne et al., 2022). TMEM119 immunostaining can be combined with IBA1 to permit distinct visualization of microglia and brain-infiltrating macrophages by immunofluorescence (van Wageningen et al., 2019). However, caution should be exercised when using TMEM119 as a selective marker for microglia. TMEM119 protein expression has been shown to be state-dependent, and can be strongly downregulated by reactive microglia in pathological conditions (Haynes et al., 2006; Segal and Giger, 2016; Bohnert et al., 2020; Vankriekelsvenne et al., 2022).

P2Y receptors are metabotropic purinergic receptors that signal in response to adenosine triphosphate (ATP), notably released by injured cells (van Wageningen et al., 2019; von Kügelgen, 2019; Gómez Morillas et al., 2021). These receptors are extensively present in microglia, but also detected in astrocytes within the CNS, in both humans and rodents (Franke et al., 2004; Milior et al., 2020; Walker et al., 2020). P2RY12 is particularly important for microglia-neuron communication, particularly through regulating microglial chemotaxis toward neuronal-derived ATP in response to injury (Haynes et al., 2006). P2RY12 serves as an effective marker for observing microglial motility, frequently revealing surveillance movements, membrane ruffling, and live imaging of process extension or retraction. In fact, *in vitro* and *in vivo* studies have shown that microglia lacking P2RY12 receptors are unable to migrate or extend processes toward nucleotides (Haynes et al., 2006; Lin et al., 2020). Microglial P2RY12 is also a key mediator of synaptic plasticity and behavior (Sipe et al., 2016; Lowery et al., 2021) and contributes to synaptic loss in models of chronic stress (Bollinger et al., 2023). Similarly to TMEM119, P2RY12 is often downregulated under inflammatory or pathological conditions (Haynes et al., 2006; van Wageningen et al., 2019; Lin et al., 2020). In post-mortem tissue samples from multiple sclerosis patients, immunoreactivity for both TMEM119 and P2RY12 was decreased in the centers of white matter lesions, which correlated with the presence of lymphocyte infiltrates and proinflammatory cytokines (van Wageningen et al., 2019). However, in a mouse model of status epilepticus, microglial P2RY12 expression and purinergic signaling were increased in the hippocampus (Avignone et al., 2008), highlighting the complex dynamics of P2RY12 expression across pathologies. Likewise, the pattern of purine-induced motility in human microglia within epileptic tissue appears to have some similarities to that observed in rodent microglia. As reported by Milior and colleagues, low doses of purine in human tissue promoted microglial branch extension, whereas in high concentrations, process retraction and membrane ruffling were facilitated (Milior et al., 2020). Notably, in the context of microglial branch extension, activation of P2Y12 was essential, mirroring that observed in rodents. Yet, the mechanism for microglial processes retraction was mediated by the activation of P2Y1 and P2Y13 receptors, rather than A2A—as classically seen in rodents (Orr et al., 2009; Milior et al., 2020). P2RY12 is extensively used in KO models, fate mapping, and reporter models driven by the purinergic signaling promoter, common within heterozygous GFP reporter mice under the fractalkine receptor promoter or TMEM119. This combination also enables *in vivo* imaging using two-photon microscopy (Bisht et al., 2021).

### Intracellular proteins and transcriptional factors

Intracellular proteins are important for vital microglial functions, such as cell organization, metabolism and adhesion, and can consistently aid in microglial visualization (Zhao et al., 2022). IBA1 is the most common cytoplasmic protein used for microglial immunostaining (Ohsawa et al., 2004; Tremblay et al., 2010). It was first isolated from rats in 1996, and labels both microglia, macrophages/monocytes and BAMs in the CNS (Imai et al., 1996; Sasaki et al., 2001; Jurga et al., 2020). IBA1 is primarily responsible for cytoskeletal reorganization, which enables processes such as membrane ruffling, migration and phagocytosis (Sasaki et al., 2001; Ohsawa et al., 2004; Hopperton et al., 2018; Sobue et al., 2021). IBA1 is commonly used in IHC, mostly in conjugation with other antibodies of interest such as TMEM119 and P2RY12, to aid the distinction of infiltrating myeloid cells, which stain dimly with these antibodies. IBA1-IHC can also be coupled to other techniques, such as CX3CR1-GFP reporter mice or single-cell approaches to describe diverse microglial subpopulations across various physiological and pathological conditions in the CNS (Ibanez et al., 2019; Lituma et al., 2021; González Ibáñez et al., 2023). In both white and gray matter, IBA1 has high sensitivity for microglia and appears to be expressed across a majority of microglial states (Shapiro et al., 2009). Moreover, IBA1 expression is found across many vertebrate and invertebrate species, highlighting its expression in humans and rodents (Swanson et al., 2020; Sharma et al., 2021). Interestingly, studies show that IBA1 expression in human microglia may be altered in pathological states, including AD (Hopperton et al., 2018). Under these conditions, microglial morphology and ultrastructure can aid in identifying microglial phenotypes. For instance, dark microglia, a phenotype identified by EM and found in mouse models and human *post-mortem* samples of AD pathology, express very low levels of IBA1 but can be distinguished by their unique ultrastructural characteristics, notably an electron-dense (dark) cytoplasm and nucleoplasm (Bisht et al., 2016). In contrast to mouse models, dark microglia observed to date in human patients with AD stain positive for IBA1 (St-Pierre et al., 2022a). Dystrophic microglia represent another morphologically-defined phenotype, staining positive for IBA1 but exhibiting thin, fragmented processes (Streit et al., 2004, 2009), and are found in brains of human AD patients in proportionately greater quantities than during normal aging (Shahidehpour et al., 2021). These microglia are thought to be senescent and dysfunctional, and their presence correlates with neuropathology in AD (Streit et al., 2009, 2020).

The advent of novel transcriptomic techniques, such as microarrays and RNA sequencing, has facilitated the identification of key microglial regulators of transcription (Olah et al., 2020). Researchers recently demonstrated that the Spalt-like 1 gene (*Sall1*) and the hexosaminidase β-subunit (*Hexb*) are selectively expressed in murine and human brain microglia and play important roles in normal microglial maturation and function (Butovsky and Weiner, 2018). In mice, SALL1 is expressed by microglia and by other mononuclear or resident glial cells of the CNS, such as astrocytes (Buttgereit et al., 2016; Chi et al., 2019). SALL1 is highly expressed in young murine CNS microglia associated with critical functions, such as neural maturation and synaptic pruning (Buttgereit et al., 2016; Holtman et al., 2017; Sobue et al., 2021; Scott et al., 2022). Microglia-specific *Sall1* deletion *in vivo* results in altered morphology and converts surveillant microglia to a reactive, proinflammatory phenotype (Buttgereit et al., 2016). Additionally, SALL1 is expressed during development and persists throughout the life of the cell, making it a useful marker for tracking microglia across the lifespan (Buttgereit et al., 2016).

Similarly, Hexb is a lysosomal enzyme that plays critical role in lysis processes, breaking down fatty compounds such as sphingolipids and gangliosides (Kuil et al., 2019). Recent studies using RNAscope and single-cell RNA sequencing have shown that Hexb is expressed in brain microglia and its expression is highly restricted to these cells within the CNS (Masuda et al., 2020; Faust et al., 2023). Notably, it was not detected in CNS-associated macrophages according to Masuda et al. (2020); however, it has been identified in non-CNS cells, such as Ly6C^+^ monocytes obtained from the bone marrow (Hickman et al., 2013; Monaghan et al., 2019; Masuda et al., 2020; Shah et al., 2022). HEXB may also play a role in pathology, with elevated gene expression of *HEXB* associated with poor prognosis in human glioblastoma (Jia et al., 2021). Although HEXB immunostaining has been utilized in combination with TMEM119 to directly identify microglia (Jia et al., 2021), *Hexb*-based gene reporter and fate mapping tools, have also recently provided important insights into the behavior and functions of microglia *in vivo* (Masuda et al., 2020). Additionally, the promoter region of *Hexb* has recently been characterized, enabling the study of *Hexb* as a potential target for microglia-targeted gene therapies in humans (Shah et al., 2022).

### Nuclear-associated markers

Classically, transcription factors modulate transcription within the nucleus, providing effective targets for identifying microglial cells. For instance, PU.1 is exclusively found in microglia in the brain, however, it is also expressed throughout the body by other cells, such as macrophages, granulocytes, and B cells (Pimenova et al., 2021; Cakir et al., 2022). PU.1 is a crucial regulator of microglial development and homeostatic function. In fact, KO of PU.1 in BV2, an immortalized mouse microglial cell line, abates most of the homeostatic microglial signatures. Conversely, it upregulates genes associated with DAMs, including *Lpl* and *Clec7a*, as assessed through q-PCR and Western Blotting in BV2-lineage cells (Pimenova et al., 2021). Moreover, elevated PU.1 expression is associated with an elevated risk of AD, whereas reduced expression is believed to offer some protective effect and contribute to improving inflammation balance by microglia, result observed in primary human mixed glial cultures following siRNA-mediated KO of PU.1 (Rustenhoven et al., 2018; Pimenova et al., 2021; Ralvenius et al., 2023). Sall1 can also be referred to as a nuclear marker for microglia. In mice, the initiation of *Sall1* expression occurs at E11 to 12 in hematopoietic progenitor cells derived from the yolk sac. These cells infiltrate the developing brain and ultimately differentiate into resident microglia. Notably, the expression of Sall1 is dependent on TGFβ1 signaling, an instrumental pathway in microglial differentiation and survival (Fixsen et al., 2023). The expression of Sall1 by microglia is context-dependent, it can decline when microglia are transplanted from the brain to an *in vitro* environment, underscoring the importance of the brain micro-environment to sustain an authentic *in vivo* microglial phenotype (Gosselin et al., 2017; Fixsen et al., 2023).

Similarly, single-cell technology has identified ZEB1 and MAFB as reliable nuclear markers for microglia. MAFB exhibits diffuse expression throughout the brain and body in both mice and humans, including neurons, microglia/macrophages, other glial cells, Kupffer cells in the liver, and Hofbauer cells, fetal origin macrophages in placenta (Anderson et al., 2023; Liu S. et al., 2023; Liu W. et al., 2023; Vondra et al., 2023). On the other hand, ZEB1 is expressed in radial glial cells and intermediate progenitor cells, as well as mature astrocytes, but seems to be downregulated in neuronal lineages (Gupta et al., 2021). Using multiomics and a methodology to delineate peak-gene-transcription factor trios in nuclei isolated from human *post-mortem* DLPFC tissues, Anderson et al. (2023) showed that the regulation of MAFB in microglia has been linked to high DAM-like signatures, including TRL3 and CD84. Interestingly, ZEB1 in neurons was identified in half of all AD-specific trios with target genes involved in regulating ion channel signaling (ITPR1, CAMK2A, CACNB3). In contrast, ZEB1 was not identified in control (non-AD) specific trios, suggesting that it may experience upregulation under pathological conditions (Maimaiti et al., 2019; Poonaki et al., 2022; Anderson et al., 2023).

### Extracellular and secreted proteins

The extracellular matrix (ECM) is a non-cellular component present in all tissues that provides a physical scaffold, biochemical and biomechanical cues, and helps organize cellular morphology and structure. Each tissue has unique ECM characteristics and structure, with specific growth factors and enzymes determining its composition and function (Theocharis et al., 2016; Lam et al., 2019). As an extracellular component, the ECM is constituted of a variety of matrix proteins that are produced by many different cell types. Components of the ECM can actively influence the adhesion, migration, and morphology of microglial cells. For instance, various components of the ECM, such as fibronectin, vitronectin and laminin, interact with microglial cell surface receptors. For instance, microglia can adhere to these specific ECM molecules through integrin receptors, reshaping microglial activity and motility according to the ECM environment (Milner and Campbell, 2002). Notably, neuronal IL 33 contributes significantly to synapse plasticity in the hippocampus of adult mice. While IL-33 guides microglial engulfment of the ECM, its deficiency results in compromised ECM engulfment, leading to the accumulation of ECM proteins in proximity to synapses (Nguyen et al., 2020). Notably, all synapses are surrounded by interstitial extracellular matrix (iECM), mostly regulating the synapse volume, however, certain synapses are enclosed by a condensed version of the ECM, known as perineuronal nets (PNNs) (Fawcett et al., 2022). Specifically, PNNs form a specialized ECM type in the CNS, providing structural support for cellular elements and maintaining synapse homeostasis. These nets are mostly formed by chondroitin sulfate proteoglycans (CSPGs) attached to a hyaluronan structure (Fawcett et al., 2022). PNNs form a net-like structure that surrounds the cell body and dendrites, enveloping synapses. It is now evident that PNNs significantly contribute to plasticity control. Interestingly, approximately 80% of parvalbumin^+^ neurons in the primary somatosensory cortex are coated with PNNs. Venturino et al. (2021) showed that microglia closely interact with parvalbumin neurons in the cortex using immunostaining against IBA1 and parvalbumin itself in adult mice. Moreover, parvalbumin neurons and IBA1^+^ microglia significantly increased matrix metalloproteinase (MMP)-9 levels upon ketamine exposure in cortical layers 3–5 (Venturino et al., 2021).

Specifically, microglia, as well as monocytes, macrophages, astrocytes and lymphocytes, produce MMPs such as MMP-3 and MMP-9, which mediate the degradation of multiple ECM components (Lindberg et al., 2001; Patnaik et al., 2021). MMPs play important physiological roles in synaptic plasticity, learning and memory (Meighan et al., 2006, 2007; Szepesi et al., 2014; Bijata et al., 2017). In fact, MMP-9 is especially vital for the formation of sensory circuits in the early postnatal period, demonstrating increased activity that aligns with synaptic reorganization during critical period plasticity and developmental windows across the CNS (Hanania et al., 2012; Reinhard et al., 2015; Shih et al., 2021; Akol et al., 2022). MMPs have been implicated in various disease states, with hippocampal induction of MMP-9 activity recently shown to mediate the development of depressive-like behavior in a mouse model of chronic stress (Bijata et al., 2022). Microglial upregulation of MMPs is also associated with inflammation or injury (Rosell et al., 2006; Colognato and Tzvetanova, 2011; Könnecke and Bechmann, 2013). Microglial-secreted MMP-3 and MMP-9 in turn influence microglia themselves, promoting production of reactive oxygen species and proinflammatory cytokines such as TNF-α and IL-1β (Kim et al., 2005; Woo et al., 2008; Könnecke and Bechmann, 2013). Recently, Kim J. et al. (2021) employed MMP-9 staining by IHC to identify microglia in the aqueous humor of patients with age-related macular degeneration and in related mouse models of choroidal neovascularization. MMP-9 expression co-localized with IBA1^+^ reactive microglia defined by an amoeboid morphology, and choroidal lesions. Notably, MMP-9 expression was suppressed by minocycline, a modulator of microglia (Kim J. et al., 2021). Thus, while the ECM itself is not specific to microglia, the use of specific ECM proteins as markers in combination with other techniques can provide a means of identifying and tracking reactive microglial states throughout physiological and pathological contexts.

## Microglia in nanoscale: electron microscopy ultrastructure analysis

**FIGURE 3:**
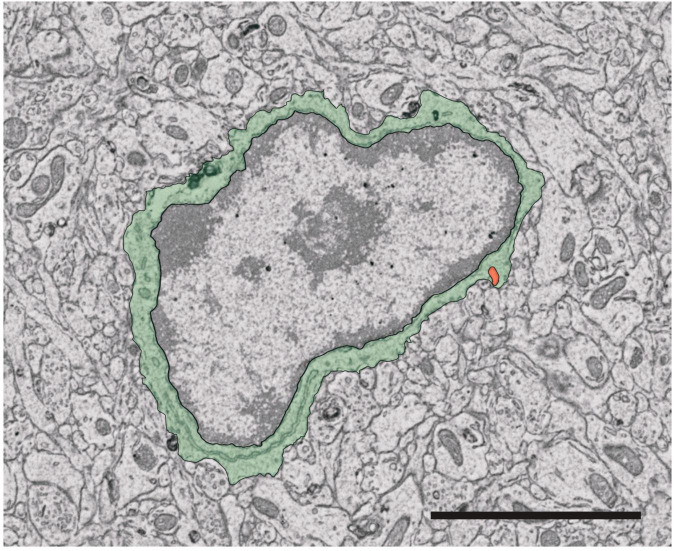
Identification of microglia for ultrastructural analysis. Microglia cell bodies in the mouse brain can be recognized based on key visual characteristics. The cell body shape varies widely between microglia but exhibits a more elongated and/or triangular shape compared to other brain cells (pseudocolored here in green). Furthermore, the cell nucleus (surrounded by the green pseudocolored cytoplasm) is characterized by two regions, heterochromatin (darker portion of the nucleus) and euchromatin (pale region of the nucleus). Certain intracellular organelles can also aid in identifying microglia, notably the presence of long stretches of endoplasmic reticulum cisternae and a phagosome (pseudocolored in orange). Scale bar: 3 μm.

The visualization of microglia using fluorescent targets has proven highly informative (Davalos et al., 2005; Nimmerjahn et al., 2005). However, the spatial resolution of fluorescence-based techniques are limited by the wavelength of the photon (Carrier et al., 2020). EM overcomes this limitation, permitting detailed examination of intracellular structures at nanometer resolution (Tremblay et al., 2010; Tremblay and Majewska, 2019; Carrier et al., 2020; Bordeleau et al., 2021). At a glance, microglia are visibly smaller than other glial cells and neurons (Nahirney and Tremblay, 2021), with the soma often less than 10 μm in diameter, and display a more triangular or elongated shape than other glial cells, but this varies depending on the tissue context (St-Pierre et al., 2022b). Microglia are also distinguished by their association with pockets of extracellular space and long stretches of endoplasmic reticulum (Tremblay et al., 2010; Nahirney and Tremblay, 2021). Microglial cell bodies are distinctive from their neuronal counterparts by a patchy “leopard” distribution of heterochromatin on the pale euchromatin background, as seen in Figure 3 (St-Pierre et al., 2022b). Additionally, the boundary of the nucleus is commonly surrounded by heterochromatin (St-Pierre et al., 2022b). Microglia in EM can sometimes be identified by the presence of intracellular inclusions such as lysosomes, lipofuscin granules, and lipid bodies (St-Pierre et al., 2022b). These cytoplasmic inclusions also provide information on the current activity of the microglia analyzed, providing insight into cellular health and function (El Hajj et al., 2019; Lecours et al., 2020; Savage et al., 2020). Interactions between microglia and neurons, particularly synapses, can be identified by directly comparing appositions of microglial cell bodies and processes with synaptic elements (Savage et al., 2020; González Ibáñez et al., 2023), while insights into phagocytosis of different cargos such as synapses can further be obtained through examining the contents of microglial intracellular inclusions (Nahirney and Tremblay, 2021). EM can be further combined with immunostaining to glean essential information about the relationship between microglial structure and function. For instance, IBA1 immunostaining is commonly employed with EM to identify microglia in the CNS (Carrier et al., 2020). Notably, dark microglia, described above, were identified based on distinctive ultrastructural characteristics in EM (Bisht et al., 2016), but their reactive state and phagocytic activity have been supported by immunostaining, which demonstrates low expression of homeostatic CX3CR1 and P2RY12 and strong immunopositivity for TREM2 (Bisht et al., 2016; St-Pierre et al., 2020). These cells are a unique state associated with multiple pathological conditions including AD mouse models and human *post-mortem* AD brain samples (St-Pierre et al., 2020, 2022a). Microglial structural analysis has also been key in defining their involvement in AD, Huntington and Parkinson’s disease pathology, as well as in chronic stress, schizophrenia and neurodevelopmental disorders (El Hajj et al., 2019; Hui et al., 2020; Lecours et al., 2020; Savage et al., 2020; Bordeleau et al., 2021; González Ibáñez et al., 2023).

## Genetically modulating microglia with reporter models

First developed in the early 1980s, gene reporter models are versatile tools to visualize, characterize and isolate specific cell populations *in vivo* (Jefferson et al., 1986, 1987; Serganova and Blasberg, 2019). Through the expression of an easily detectable fluorescent reporter protein under the control of a microglia-specific promoter, reporter genes have provided key insights into the dynamics and activities of these cells (Jiang et al., 2008). Additionally, reporter models can be used to investigate the roles of microglia in various physiological and pathological processes, and to characterize the effects of genetic mutations or disease states on microglial functions (Nonnenmacher and Weber, 2012; Serganova and Blasberg, 2019).

### Constitutive reporter models for microglial study

The advent of homologous recombination-based techniques has allowed researchers to place a reporter gene of interest into a microglia-specific locus, replacing the endogenously expressed allele. This has been used effectively to create mouse models expressing fluorescent reporters in place of myeloid or microglia-specific proteins such as CX3CR1 (Jung et al., 2000). Constitutive reporter models have proven immeasurably useful for the visualization, identification, and study of microglia through the targeting of countless microglial genes such as *Cx3cr1* (Garcia et al., 2013), *Tmem119* (McKinsey et al., 2020; Ruan et al., 2020), *Sall1* (Buttgereit et al., 2016; Baba et al., 2019) and *Hexb* (Masuda et al., 2020). The intrinsic fluorescence of reporter molecules such as eGFP and tdTomato (tdT) eliminates the need for IHC and other techniques requiring cell fixation, allowing researchers to gain insights into microglial interactions with their surroundings in an *in vivo* context. However, a disadvantage to this approach is the potential presence of mutations incurred by the random insertion of the construct into the genome (Wieghofer et al., 2015).

Modern approaches extensively employ the Cre/loxP system, which allows cell type-specific gene inactivation or activation (Orban et al., 1992). In this paradigm, the bacteriophage-derived Cre recombinase is expressed under cell-specific control, and precisely and irreversibly excises a distal sequence of DNA flanked by upstream and downstream loxP sequences, known as the “floxed” sequence (Orban et al., 1992; Wieghofer et al., 2015). Cre can be expressed in a microglial locus, for instance, under the *Cx3cr1* promoter (Parkhurst et al., 2013; Yona et al., 2013), in mice engineered to harbor a construct consisting of a strong constitutive promoter, often *Rosa26*, followed by a floxed STOP codon preceding a reporter gene such as *YFP* (abbreviated *R26^YFP^*) (Friedrich and Soriano, 1991; Wieghofer et al., 2015). In this example, Cre expression is governed by the endogenous regulation of *Cx3cr1* and is therefore constitutively expressed in microglia and other myeloid cells that normally would highly express *Cx3cr1* (Parkhurst et al., 2013; Yona et al., 2013). In CX3CR1^+^ cells, Cre excises the floxed STOP codon, leading to constitutive expression of the reporter under the control of the strong promoter. Hence, CX3CR1^+^ cells are labeled with the reporter gene of interest, enabling, for example, their identification via fluorescence microscopy or flow cytometry, or FACS-mediated isolation. Despite their utility, constitutive reporter models are limited by their property of labeling all the cells that express the gene of interest at any point during development, as Cre-mediated excision of the floxed STOP codon is irreversible. This leads to off-target cell labeling, potentially confounding results. For example, neurons transiently express *Cx3cr1* during development and are labeled in *Cx3cr1^Cre^*-based constitutive models (Haimon et al., 2018; Zhao et al., 2019), highlighting the need to interpret the results of such models cautiously. This issue is resolved with inducible reporter systems, discussed below. Reporter models to study microglia are summarized in Table 2.

**TABLE 2:** Mouse models commonly used in microglial fate visualization, depletion and fate mapping.

Target gene	Major mouse models	CNS expression and microglial specificity	Applications	Limitations	References
*Clec7a*	*Clec7a* ^*KO*^	Expressed in microglia and CNS-infiltrating myeloid cells.	Study of microglia in neuroinflammation and neurodegeneration, depletion.	KO is non-specific to microglia, with CNS functional effects likely mediated by infiltrating myeloid cells in contexts of inflammation.	Deerhake et al., 2021; Wang et al., 2022
*Csf1r*	*Csf1r*^*KO*^, *fms*-intronic regulatory element deletion (ΔFIRE)	CSF1R is broadly expressed across microglia/macrophages. ΔFIRE can selectively deplete microglia in the CNS.	Fate mapping in development, depletion.	Broad deficits brain development and function, bone structure in *Csf1r^–/–^* mice, which do not survive to adulthood.	Ginhoux et al., 2010; Erblich et al., 2011; Elmore et al., 2014; Gomez Perdiguero et al., 2015; Rojo et al., 2019; Pons et al., 2021
*Cx3cr1*	*Cx3cr1-eGFP*, *Cx3cr1*^*YFP–CreER*^, *Cx3cr1*^*Cre*^, *Cx3cr1*^*CreER*[*ERT*2]^, *Cx3cr1/Sall1-Split Cre*, *Cx3cr1*^*KO*^, Microfetti	Also expressed in BAMs and other CNS-associated macrophages. Expressed transiently in neurons during development.	Visualization, fate mapping, microglia-neuron interactions, depletion.	High spontaneous recombination (increases with mouse age; up to ∼22% in 6-month-old mice), reduced expression of endogenous *Cx3cr1*, labeling of neuronal subsets in constitutive reporter models.	Jung et al., 2000; Garcia et al., 2013; Parkhurst et al., 2013; Yona et al., 2013; Goldmann et al., 2016; Tay et al., 2017; Haimon et al., 2018; Zhao et al., 2019; Chappell-Maor et al., 2020; Van Hove et al., 2020; Faust et al., 2023
*Hexb*	*Hexb* ^*CreERT*2^	Specific to microglia within the CNS; consistently expressed across homeostasis and pathology.	Fate mapping.	Recombination efficiency ∼34% (heterozygous) or ∼62% (homozygous), decreased endogenous HEXB activity.	Masuda et al., 2020; Faust et al., 2023
*Kit*	*Kit^MerCreMer^*	Expressed in yolk sac erythromyeloid progenitors, which give rise to microglia and some Langerhans cells.	Microglial fate mapping in development (macrophage fate mapping in adulthood/pathology).	Labeling of microglia restricted to specific window between E7.5-E9.5. Labels BAMs and CNS-associated monocytes/macrophages in adult mice.	Sheng et al., 2015; Wu et al., 2021
*P2ry12*	*P2ry12^CreER^*	Specific to microglia within the CNS.	Visualization, fate mapping.	Recombination efficiency ∼25% (heterozygous), downregulated by tamoxifen. Altered expression during inflammation.	McKinsey et al., 2020; Faust et al., 2023
*Prom1*	*Prom1* ^*CreERT*2^	Expressed by a subset of stem-like microglial progenitors	Fate mapping in adult homeostasis.	Prom1^+^ progenitors account for only a fraction of newly generated microglia; limited utility in models of pathology.	Prater et al., 2021
*Runx1*	*Runx1^MerCreMer^*	Expressed by yolk sac progenitors, which give rise to microglia	Fate mapping of microglia during development.	Expressed transiently in embryonic development and does not label precursors originating at later timepoints; some off-target labeling of yolk sac cells.	Ginhoux et al., 2010; Sheng et al., 2015; O’Koren et al., 2016
*Sall1*	*Sall1*^CreER^* Cx3cr1/Sall1-Split Cre*	Not expressed by most other CNS macrophages, except a small population of choroid plexus-associated BAMs. Expressed in subsets of oligodendrocytes, astrocytes, neurons.	Visualization, fate mapping.	Spontaneous recombination. Downregulated in pathological states.	Buttgereit et al., 2016; Chappell-Maor et al., 2020; Van Hove et al., 2020; Kim J.-S. et al., 2021
*Tmem119*	*Tmem119* ^*CreERT*2^	Recombination observed in endothelial cells during development; border-associated ALDH1A2^+^/S100A6^+^ fibroblasts, leptomeningeal macrophages.	Visualization, fate mapping.	Recombination efficiency ∼50% (heterozygous) or 66% (homozygous), decreased endogenous TMEM119 expression. Downregulated in pathological states.	Kaiser and Feng, 2019; McKinsey et al., 2020; Ruan et al., 2020; Faust et al., 2023
*Trem2*	*Trem2^KO^, Trem2^KO^:5XFAD*	Expressed by microglia within the CNS, BAMs and CNS-infiltrating myeloid cells. It includes microglial phagocytic phenotypes such as DAM and dark microglia.	Studies of microglial phagocytosis, synaptic pruning, DAM.	Present only in certain microglial states.	Wang et al., 2015, 2022; Keren-Shaul et al., 2017

### Inducible reporter models for microglial study

More recently, the advent of inducible Cre-based systems has revolutionized the field, enabling precise temporal labeling of specific cell populations and their progeny. This approach makes use of a modified estrogen receptor (ER, MER, ERT, or ERT2), which can bind the estrogen analog 4-hydroxytamoxifen but not endogenous estrogens (Littlewood et al., 1995; Metzger et al., 1995; Feil et al., 1997; Indra et al., 1999). Development of CreER or CreERT2 fusion proteins has enabled researchers to control when Cre expression is induced, thus allowing temporal-specific induction of reporter gene expression (Feil et al., 1997; Hayashi and McMahon, 2002). Since Cre-mediated excision of the floxed sequence is irreversible, reporter gene expression is maintained in all subsequent daughter cells following mitosis, enabling the tracking of specific cell populations and their progeny (Hayashi and McMahon, 2002; Wieghofer et al., 2015). Drawing on our earlier example of *Cx3cr1*, placing *CreER* under the control of *Cx3cr1* (*Cx3cr1^CreER^*) and administering tamoxifen at a specific time in the mouse lifespan would induce constitutive reporter gene expression in all cells expressing *Cx3cr1* at that timepoint, as well as in all progenies of those cells. This critical property of inducible Cre systems has allowed researchers to selectively deplete microglia, to precisely map microglial populations in a spatiotemporally controlled manner, and to identify key activities of microglia during development, homeostasis, and pathology (Zhao et al., 2019; Dai et al., 2022; Sahasrabuddhe and Ghosh, 2022), as discussed in the following sections.

However, it is important to note that inducible microglia-targeted CreER lines vary considerably in their stability, with a recent analysis showing that *Cx3cr1^CreER^* tools demonstrate particularly high rates of spontaneous recombination in the absence of tamoxifen, compared to *Tmem119^CreER^*- or *Hexb^CreER^*-based lines (Faust et al., 2023). The authors also demonstrated, in some cases, reduced expression of the endogenous gene under the promoter driving *CreER* expression (Faust et al., 2023); as the genes used often encode essential microglial proteins, this could impair the function of Cre-targeted microglia and confound results. Although microglial gene expression is largely unaffected by tamoxifen, it was recently demonstrated that tamoxifen administration can itself lead to lower protein expression of P2RY12 (Faust et al., 2023). These issues represent important considerations when interpreting data from Cre-based microglial reporter, KO, and fate-mapping models.

Cre-based reporter systems are inherently limited by the specificity of the target gene for the cell type of interest. For example, inducible *Cx3cr1*-based systems, commonly used to target microglia, also affect CNS-associated and perivascular macrophages (Haimon et al., 2018; Masuda et al., 2020; Faust et al., 2023). To overcome this limitation, binary “split Cre” transgenic systems have been developed, requiring the cell of interest to simultaneously express two separate fragments (NCre and CCre) of the Cre recombinase enzyme placed under the control of different cell-specific promoters, which dimerize into a functional protein only when co-expressed (Jullien et al., 2003; Hirrlinger et al., 2009). In the context of microglia, this approach was leveraged using the clustered regularly interspaced short palindromic repeats (CRISPR)/Cas9 system to co-express NCre and CCre under the control of *Cx3cr1* and *Sall1*, selectively targeting *Cx3cr1*^+^/*Sall1*^+^ microglia, but not *Cx3cr1*^+^/*Sall1*^–^ infiltrating or vasculature-associated myeloid cells (Kim J.-S. et al., 2021). This enabled differential analysis of microglial and BAMs translatomes through a Cre-mediated RiboTag strategy (Kim J.-S. et al., 2021), highlighting strong potential of binary Cre systems for future use in high-specificity studies of microglial biology.

## Microglial ablation: genetic and pharmacological approaches

As discussed, several methods can be used to achieve microglial visualization and provide a more complete understanding of these cells in the CNS (Tang et al., 2015). However, by depleting microglia, researchers can directly assess their role in a particular physiological or pathophysiological process by observing the consequences of their removal through targeted genetic and pharmacological ablation methods (Saxena et al., 2021).

The most target-specific depletion methods are gene KO models achieved by homologous recombination or, more recently, CRISPR-based genome editing, to excise a specific microglial gene (Albadri et al., 2017; Wang and Sun, 2019; Ayanoğlu et al., 2020; Damasceno et al., 2020; Krais and Johnson, 2020; Zuccaro et al., 2020). Although genome editing techniques offer many advantages, inherent limitations include the potential for instability at the altered site, as well as the possibility of unintended insertions and deletions (Doudna and Charpentier, 2014; Wang and Sun, 2019). On the other hand, pharmacological ablation allows the use of chemicals to eliminate or reduce the function of microglia. These drugs can be designed to block the activity of specific proteins or signaling pathways that are important for microglial survival or function (Stojiljkovic et al., 2022). It should be noted that these techniques are not without flaws, and their specificity and efficacy may vary depending on the context and the model used (Han et al., 2017). One important caveat to depletion methods that kill living microglia is their potential to exert non-specific effects on other CNS-cell types, such as astrocytes or BAMs, in response to dead or dying microglial cells (Elmore et al., 2014; Unger et al., 2018; Marino Lee et al., 2021).

Diverse proteins have been targeted for the depletion of microglia, including markers previously discussed in the context of visualization. The following section will focus on highlighting additional proteins that may be useful for this purpose.

### Genetic depletion of microglial subsets

As a result of advances in gene editing techniques, methods of microglial depletion—based on KO animal models of essential genes in microglial survival and function—have provided a more comprehensive understanding into the roles of microglia in development, homeostasis, and pathology. Meanwhile, selective KO models of microglial genes across various contexts have helped us better understand the roles of specific genes and microglial states dependent on those genes.

Microglia, as well as monocytes and other macrophage populations, highly express the receptor for colony-stimulating factor-1 (CSF1R). CSF1R signaling through its ligands, CSF-1, and IL-34 (Wang et al., 2012), is essential for microglial development, survival, proliferation and release of proinflammatory factors (Mitrasinovic et al., 2005; Ginhoux et al., 2010; Erblich et al., 2011; Elmore et al., 2014; Green et al., 2020). As a result, CSF1R is a well-established target for microglial depletion. The loss of CSF1R in *Csf1r*^–/–^ mice leads to nearly a complete lack of microglia, with > 99% depletion at E16 and postnatal day 1 (Ginhoux et al., 2010; Erblich et al., 2011). However, *Csf1r*^–/–^ mice also show deficits in brain size and function, as well as some deficiencies of the olfactory bulb development, myelination and bone structure, leading to a premature death (Erblich et al., 2011; Green et al., 2020). It thus requires the use of inducible Cre/lox systems or pharmacological inhibitors of CSF1R (discussed below) to selectively deplete microglia in adult mice. Pons et al. (2021) used the Cre/loxP system to delete CSF1R in 3-month old mice, achieving a KO efficiency of 89%, and showed that loss of *Csf1r* in adulthood did not impact microglial survival. Intriguingly, deletion of *Csf1r* in adult APP-PS1 mice significantly ameliorated AD amyloid-beta pathology (Pons et al., 2021), highlighting the potential of this tool to elucidate the complex roles of microglia in neurodegenerative disease. Deletion of *Csf1r* regulatory elements, such as the *fms*-intronic regulatory element (FIRE), can also deplete microglia by selectively abolishing *Csf1r* expression in a cell- and tissue-specific manner (Rojo et al., 2019). Mice with CRISPR-mediated homozygous FIRE deletions completely lack microglia by IBA1 and P2RY12 immunostaining, as well as several other tissue-resident macrophage populations, but otherwise develop into healthy fertile adults without the systemic defects observed in *Csf1r*^–/–^ mice. Notably, flow cytometry identified retention of CD11b^+^/CD45^hi^ brain macrophages, which also co-expressed the perivascular macrophage marker CD169 by immunostaining (Rojo et al., 2019), suggesting a high degree of depletion specificity to microglia. Researchers have since crossed FIRE KO mice with the 5XFAD mouse model to study the effects of selectively lacking microglia in AD pathology, showing that the absence of microglia ameliorated amyloid-beta pathology but worsened cerebral amyloid angiopathy, which could be prevented by intra-hippocampal transplantation of wild-type microglial cells (Kiani Shabestari et al., 2022). The use of highly selective microglial depletion models holds great promise to elucidate microglial roles, and associated therapeutic targets, across diverse pathologies.

As previously discussed, the CX3CR1 receptor is a powerful tool to investigate neuron-microglia communication (Wolf et al., 2013), and *Cx3cr1* KO models have been critical for our understanding of the fractalkine signaling pathway. Pagani et al. (2015) demonstrated that microglia-specific *Cx3cr1* KO leads to acute deficits in the developing hippocampus, including impairments in the response to extrinsic ATP and altered electrophysiological properties. In mouse models of chronic stress, *Cx3cr1* deficiency attenuated stress-induced alterations to microglial morphology and reduced phagocytosis (Milior et al., 2016), highlighting a key role for microglia-neuron communication in the response to stress. An additional method microglial depletion is achieved by placing the diphtheria toxin receptor (DTR) under the control of a microglial promoter using inducible Cre-loxP models, allowing for an efficient depletion when diphtheria toxin is administered. Using *Cx3cr1^CreER^:R26^iDTR^* mice, Parkhurst et al. (2013) selectively depleted *Cx3cr1*-expressing microglia to show a critical role for these cells in brain-derived neurotrophic factor-dependent learning and synaptic plasticity. A recent study used similar tools to assess the effects of depleting *Cx3cr1*^+^ microglia on kainic acid-induced status epilepticus (Wu et al., 2020). Depletion of microglia through inducible expression of diphtheria toxin, DTR or KO of the essential *Csf1r* in *Cx3cr1*^+^ cells, led to exacerbated disease pathology, increased mortality and enhanced neurodegeneration (Wu et al., 2020). However, as *Cx3cr1* is also expressed on infiltrating and border-associated myeloid cells, future studies would benefit from employing more selective depletion tools such as binary Cre-based systems targeting multiple co-expressed microglial genes (Kim J.-S. et al., 2021).

Triggering receptor expressed on myeloid cells-2 (TREM2) is essential for microglial synaptic pruning and formation of normal neural circuitry during brain development by mediating phosphatidylserine-dependent phagocytosis of apoptotic neurons (Filipello et al., 2018; Scott-Hewitt et al., 2020). It is also involved in the microglial response to injury and in the recognition of soluble factors (Quan et al., 2009; McQuade et al., 2020). Microglial TREM2 is heavily implicated in disease and is required for the transformation into DAM, MgND and other pathology-associated states (Keren-Shaul et al., 2017; Krasemann et al., 2017; Deczkowska et al., 2018). TREM2 is selectively expressed on microglia within the CNS, but is found on other myeloid-derived cells such as macrophages/monocytes, dendritic cells and granulocytes in the periphery (Yaghmoor et al., 2014; Gratuze et al., 2018). Soluble forms of TREM2 increase microglial survival and proliferation and induce morphological changes during inflammatory responses (Zhong et al., 2017). Furthermore, *TREM2* variants in humans have been strongly implicated in AD risk (Guerreiro et al., 2013; Jonsson et al., 2013; Sims et al., 2017). Genetic *Trem2* deletion models have greatly contributed to our knowledge of its normal functions and implications in microglial biology, generally implicating TREM2 as a critical mediator of phagocytosis in development and pathological states. *Trem2*^–/–^ mice show decreased microglial numbers in the CA1 region of the hippocampus with reductions in reactive microglial morphologies, increased synaptic density and overactive excitatory neurotransmission, highlighting the importance of *Trem2* in the removal of extraneous synapses during neural development (Filipello et al., 2018). These deficits are recapitulated in human iPSC-derived microglia *in vitro*, which following CRISPR-mediated deletion of *TREM2*, are unable to phagocytose apolipoprotein E or fibrillar amyloid-beta, and present impaired survival in response to stress (McQuade et al., 2020). In line with this, 5XFAD mice deficient in *Trem2*, or its downstream mediator *Syk*, display an impaired amyloid-beta plaque clearance by microglia (Wang et al., 2015, 2022), and do not develop DAM (Keren-Shaul et al., 2017; Wang et al., 2022). Similarly, in a humanized mouse model of Tau pathology, crossed mice lacking *Trem2* presented augmented Tau aggregation and phosphorylation with altered morphology in IBA1^+^ microglia by IHC (Bemiller et al., 2017). TREM2 is also expressed on plaque-associated dark microglia by EM in the APP-PS1 model of AD pathology (Bisht et al., 2016), suggesting a role for dark microglia in amyloid-beta phagocytosis. However, selective deletion of *Trem2* in microglia leads to an altered transcriptional profile and phenotype, temporarily improving disease in mouse models of traumatic spinal cord injury and experimental autoimmune encephalomyelitis (Gao et al., 2023). Overall, *Trem2* KO models have enabled the selective depletion of pathologically relevant microglial populations, shedding light on the diverse roles of microglia in disease.

In a similar fashion, C-type lectin domain family member 7a (CLEC7a), also known as DECTIN-1, is expressed on cells of the monocyte lineage and functions in the recognition and phagocytosis of fungal and bacterial pathogens (Brown et al., 2002). CLEC7a was also shown to play a role in cancer progression through its influences on tumor-associated macrophages (Daley et al., 2017). CLEC7a is expressed in microglia, where it promotes phagocytosis upon ligation (Shah et al., 2008). CLEC7a signaling directly activates SYK-mediated pathways downstream of TREM2, enhancing phagocytosis and representing a potential therapeutic target for individuals with deficits in TREM2 function (Wang et al., 2022). In mouse models of AD pathology, brain *Clec7a* is significantly upregulated in DAM and MgND states (Keren-Shaul et al., 2017; Krasemann et al., 2017; Deczkowska et al., 2018). CLEC7a is also upregulated by microglia in mouse models of experimental autoimmune encephalomyelitis (Krasemann et al., 2017; Deerhake et al., 2021), with *Clec7a*^–/–^ mice displaying more severe disease, although these effects were more likely mediated by CNS-infiltrating myeloid cells than microglia (Deerhake et al., 2021). These examples highlight the potential for CLEC7a KO models to continue to enhance our understanding of microglial roles in disease conditions.

### Pharmacological depletion of microglia

The pharmacological depletion of microglia in the CNS has been a valuable and accessible tool in order to define their role across contexts of health and disease, with the advantage of being less challenging than generating and utilizing a KO model (Barnett et al., 2021). This has been made possible due to a vast array of compounds that demonstrate the capacity to modulate microglial numbers and control their activities, notably in mediating CNS inflammation (Barnett et al., 2021).

As discussed, the use of CSF1R inhibitors is an emerging technique with demonstrated efficacy in the ablation of microglia in the CNS (Elmore et al., 2014; Fujiwara et al., 2021). The CSF1R inhibitors, such as PLX3397 and PLX5622, are CNS-permeable and selectively inhibit tyrosine kinase receptors on macrophages and microglia, although the specificity of these inhibitors varies depending on the specific compound used (Green et al., 2020; Hupp and Iliev, 2020), with PLX5622 recently shown to exert effects on peripheral bone marrow and tissue-resident macrophages (Lei et al., 2020). CSF1R inhibitors are orally bioavailable, but their routes of administration can also include intravenous or subcutaneous injection (Hupp and Iliev, 2020). Moreover, CSF1R inhibitors have been shown to reduce microglial proliferation, depleting the majority of the brain population (Elmore et al., 2014; Acharya et al., 2016; Fu et al., 2020). It has been reported that most of IBA1^+^ microglia are eliminated within the first week of administration—as assessed with staining against caspase-3, a marker for cell apoptosis. Approximately 90% of microglia can be eliminated following 7 days of PLX3397 treatment, shown using flow cytometry in whole brains of CX3CR1-GFP^+/–^ adult mice (Elmore et al., 2014). Effects remain with sustained administration of CSF1R inhibitors—for instance, after 21 days of PLX3397 treatment (Elmore et al., 2014). However, microglial repopulation is notable between drug withdrawal and the 3-day recovery time point. This highlights the critical period of 48–72 h for microglial repopulation after CSF1R inhibition (Elmore et al., 2014; Henry et al., 2020; Shi et al., 2022). PLX3397 has undergone exploration in humans; however, its efficacy has not yet achieved that observed in mice. In a phase II clinical trial for recurrent glioblastoma, a daily oral dose of 1,000 mg of PLX3397 was well-tolerated and successfully crossed the blood-brain barrier of 37 patients (mean age of 58.5; 25 males and 12 females). Nevertheless, it demonstrated no effectiveness in reducing tumor size or microglial density within the tumor tissue. IHC for IBA1 was conducted and used to quantify cell numbers and staining intensity. No statistically significant differences were observed in IBA1^+^ microglia between pre-study (archival) and on-study (surgical) samples, whether treated with PLX3397, following the standard of care with radiation therapy complemented with temozolomide chemotherapy, or when compared to historical control samples (Butowski et al., 2016).

Interestingly, the elimination of microglia using CSF1R inhibitors was reported to prevent amyloid-beta plaque formation and disease progression in the 5XFAD and APP-PS1 mouse models of AD pathology (Olmos-Alonso et al., 2016; Spangenberg et al., 2019), and to reduce accumulation of pathogenic Tau in the Tg2541 tauopathy model, extending survival of female but not male mice (Johnson et al., 2023). Likely microglia, BAMs have the fully capacity to repopulate via local self-renewal after depletion via PLX3397 (Van Hove et al., 2019). These studies highlight CSF1R as a potential target to deplete microglia in therapeutic approaches for neurodegenerative diseases.

Minocycline, a drug initially developed to treat bacterial infections, has been shown to control and deplete inflammatory cells in the CNS (Garrido-Mesa et al., 2013; Scholz et al., 2015). Recently, it was reported that tetracyclines not only display anti-microbial activity, but also act as anti-inflammatory and anti-apoptotic agents while impairing proteolysis and angiogenesis (Bernier and Dréno, 2001; Scholz et al., 2015). Moreover, minocycline has emerged as a potential neuroprotective agent in experimental models for several CNS disorders, such as brain ischemia (Yrjänheikki et al., 1998), brain injury (Sanchez Mejia et al., 2001), Parkinson’s disease (Du et al., 2001; Thomas and Le, 2004), Huntington disease (Chen et al., 2000) and AD (Choi et al., 2007). Notably, minocycline appears to exert an anti-inflammatory influence on microglia (Bassett et al., 2021; Long et al., 2023). To exemplify, in a mouse model of subarachnoid hemorrhage (SAH), minocycline treatment was administered daily until day 7 post-SAH and continued every second day until day 14. IHC for IBA1 and a functional phagocytosis assay, employing glucose-coated beads cross-linked with a lipid bilayer and co-localized with IBA1^+^ cells in brain slices, indicated a significant reduction in the phagocytic activity of microglia/macrophages. This reduction was associated with decreased spatial interaction with neurons (NeuN^+^) and a decline in neuronal apoptosis (TUNEL^+^/NeuN^+^) (Blecharz-Lang et al., 2022).

The efficiency of minocycline in depleting microglia can vary depending on the specific conditions of the study, such as the dosage, route of administration and duration of treatment, however, overall it has been shown to be a relatively effective method to modulate microglial populations, typically achieving 50–90% depletion (Blecharz-Lang et al., 2022). However, minocycline also exhibits broad off-target effects in the CNS, and thus results from minocycline-based depletion studies should be considered with caution (Strahan et al., 2017).

Likewise, clodronate-containing liposomes (Clod-Lips) constitute an effective approach to deplete macrophage-like cells across tissues (Rooijen and Sanders, 1994; Moreno, 2018). Initially discovered as an osteoclast inhibitor, clodronate, following phagocytosis and entry into the macrophage cytosol, perturbs iron metabolism and inhibits mitochondrial respiration through its actions as an ATP analog, leading to apoptosis (Lehenkari et al., 2002; Moreno, 2018; Opperman et al., 2019). The efficiency of clodronate depends on its route of administration; intrarectal and intraperitoneal administration deplete around 50% of the macrophage population, while 90% can be achieved through the intravenous route (Weisser et al., 2012). A major drawback to Clod-Lips is their requirement for direct infusion into the CNS, as well as their demonstrated off-target effects on blood vessels (Han et al., 2019). Similarly, Takagi et al. (2020) demonstrated that Clod-Lips markedly attenuated the activation of mouse brain circuits for TLR2-mediated inflammation in a hypothermia mouse model.

Mac-1-saporin (Mac-1-sap), a chemical conjugate of a CD11b monoclonal antibody and the ribosome-inactivating protein saporin, is another pharmacological option for microglial ablation that has been shown to deplete CD11b^+^ cells in mouse models (Acharjee et al., 2018). Although not specific to microglia, targeting all CD11b^+^ cells, intrathecal injection of Mac-1-sap depletes around 50% microglia in the CNS (Yao et al., 2016). Following depletion, repopulating microglia were shown to respond to injury, re-establishing normal function after ablation (Yao et al., 2016). *In vitro* work further investigated the depletion of microglia from mouse hippocampal cultures in a model of ischemia-like oxygen-glucose deprivation. In this context, seven-day treatment with Mac-1-sap drove neural apoptosis and astrogliosis one day after oxygen-glucose deprivation (Montero et al., 2009), supporting the importance of microglia in the response to ischemic injury.

## Fate mapping: tracking microglia through development, health, and disease

In general, fate mapping approaches aim to mark specific subsets of cells at a given point in time, allowing researchers to track the spatial and temporal distribution, roles, and dynamics of these cells and their progeny. Early work by Lawson et al. (1992) employed radioactive [H-3]-thymidine pulses, observing that F4/80^+^ microglia incorporated labeled nucleotides to synthesize new DNA and proliferated to produce new microglia *in situ*. Modern experiments have used similar pulse-chase approaches to characterize microglial proliferation and turnover under steady-state conditions, showing that microglia are a dynamic, actively self-renewing population (Askew et al., 2017). However, most fate mapping studies approaches have focused on mouse models that genetically target microglia, with molecular markers increasing in both sensitivity and specificity over the years in tune with our rapidly advancing understanding of microglial gene expression profiles (Tay et al., 2017). Recent advances in microglia-specific genetic fate mapping technologies have greatly enhanced our understanding of microglial origin, dynamics in development and homeostasis, and functions in pathology. Importantly, modern fate mapping studies have enabled researchers to precisely disentangle the roles of microglia from other myeloid populations in the CNS and have revealed great heterogeneity within the microglia themselves.

### Microglial fate mapping during development

Several important microglial fate mapping tools were developed specifically aiming to decipher the origin of microglia. In these approaches, early embryonic progenitor cells are labeled based on specific gene expression patterns during development, and their progeny is followed to identify the progenitor cells giving rise to microglia and the key transcription factors regulating these processes. In a landmark study, Ginhoux et al. (2010) used fate-mapping techniques to determine that microglia develop from yolk sac-derived myeloid progenitors that arise prior to E8 in mice. In this study, Cre was co-expressed with Runx1, a transcription factor expressed in early hematopoietic progenitors, in mice harboring a *Rosa26-fl-STOP-fl-YFP* construct (*Runx1^MerCreMer^:R26^YFP^*). Tamoxifen administration at E7.5 labeled all subsequent microglia as YFP^+^, demonstrating that microglia develop from yolk sac-derived *Runx1*-dependent progenitors (Ginhoux et al., 2010). This finding was later confirmed using a similar approach (Hoeffel et al., 2015), and Runx1-based fate mapping was again employed to show that retinal microglia share a common yolk-sac origin with brain microglia (O’Koren et al., 2019). Additional research further described microglial development by co-expressing CreER with CSF1R, a constitutively expressed receptor in microglia, monocytes, and macrophages, in R26^YFP^ mice. Using tamoxifen administration to induce YFP labeling at different time points in development further revealed that microglia develop independently from bone-marrow hematopoietic stem cells (Schulz et al., 2012; Gomez Perdiguero et al., 2015; Hoeffel et al., 2015). Another study took advantage of the transcription factor Kit, expressed in early yolk sac progenitors, using *Kit^MerCreMer^:R26^YFP^* mice to show that only microglia, along with a subset of Langerhans cells, develop from early (E7.5) Kit^+^ erythromyeloid progenitors in the yolk sac (Sheng et al., 2015). Thus, microglial fate mapping tools have been crucial to building our current knowledge of microglia as an ontogenetically distinct macrophage lineage.

### Microglial fate mapping during adult homeostasis

Several key questions in modern microglial biology relate to dissecting the roles of microglia from other CNS myeloid cells and distinguishing among heterogeneous states within the microglia. Fate mapping studies have been critical in addressing these fundamental concepts. O’Koren et al. employed a combination of fate mapping and 12-color flow cytometry to definitively distinguish between retinal microglia and monocyte-derived macrophages, which both express CX3CR1, in *CX3CR1^YFP–CreER^:R26^RFP^* mice. Following tamoxifen administration, both lineages expressed RFP, however, short-lived monocyte-derived macrophages were replaced by RFP^–^ cells over a subsequent “washout” period, while long-lived microglia retained expression of RFP (O’Koren et al., 2016). Using a similar mouse model, the time course of microglial and macrophage infiltration into the various compartments of the eye was recently mapped at high spatiotemporal resolution (Wieghofer et al., 2021). More recently, researchers identified prominin 1 as a marker of committed myeloid progenitors in the adult CNS under homeostatic conditions, and employed a novel fate mapping approach using *Prom1*^*CreERT*2^*:R26^tdT^* mice to demonstrate that Prominin-1 (Prom1)^+^ progenitors also contribute to microglial proliferation during homeostasis (Prater et al., 2021).

Heterogeneity within the microglial population is an emerging concept that, until recently, could not be explored with available fate-mapping tools. To address this issue, Tay et al. (2017) created the “Microfetti” mouse strain, a cross between the existing *CX3CR1^CreER^* line and a *R26^Confetti^* strain, which contains a four-color reporter system. In Microfetti mice, tamoxifen administration causes random expression of one of four fluorescent reporters in each cell thus creating up to ten distinct color combinations in the homozygous state (Tay et al., 2017). Using mathematical models to evaluate the likelihood of spatial association of same-color cells due to chance versus cell division, the authors achieved detailed tracing of microglial cells and their progeny at single-cell resolution. This revolutionary approach revealed that microglial proliferation is a stochastic process governed by regional environmental cues during homeostasis (Tay et al., 2017).

### Microglial fate mapping during pathology

Microglia, as the innate immune cells of the CNS, play diverse and complex roles in the response to tissue injury, infection and in chronic pathological states. Fate mapping studies have allowed researchers to discern the distinct roles of microglia, BAM subsets, and infiltrating peripheral myeloid cells. For example, the source of repopulating microglia following experimental CSF1R-mediated depletion has been extensively debated. Huang et al. (2018) employed a fate mapping approach to address this issue. Using *CX3CR1*^*CreERT*2^*:R26^tdT^* mice given tamoxifen early in postnatal life, they showed that all repopulating cells were tdT^+^ and were thus exclusively derived from surviving microglia, rather than peripheral monocytes (Huang et al., 2018). Subsequent research using a similar fate mapping model has traced these repopulating microglia to a MAC2^+^ progenitor population with an immature gene expression signature that displayed resistance to CSF1R inhibition (Zhan et al., 2020). Other groups have employed comparable techniques to replicate these results in the retina (Zhang et al., 2018; O’Koren et al., 2019).

Single-cell-resolution fate mapping, using the *CX3CR1^CreER^:R26^Confetti^* model described above, showed that microglia proliferate in waves of clonal expansion under pathological conditions, and that homeostasis is restored through both migration away from the site of pathology and apoptosis (Tay et al., 2017). A recent study mapped the dynamics of microglial populations in an inducible mouse model of AD pathology (Wu et al., 2021). Using a *Kit^MerCreMer^:R26^YFP^* system with tamoxifen-mediated YFP induction periodically over 8 months, microglia, BAMs and parenchymal macrophages were shown to remain relatively stable throughout disease progression, while other myeloid cells were rapidly and continuously replaced by bone marrow-derived monocytes (Wu et al., 2021). In cuprizone-induced demyelination, fate mapping using *CX3CR1^CreER–iresGFP^:R26^dsRed^* mice also revealed that microglia, not bone marrow-derived macrophages, are the primary cells involved in the remyelination response (Marzan et al., 2021).

Fate mapping in mouse models of retinal pathology have also provided intriguing findings. In a *CX3CR1*^*CreERT*2^*:R26*^*tdT*^ mouse model of retinal ischemia-reperfusion, proliferation of resident microglia and recruitment of peripheral monocytes were found to play similarly important roles in the inflammatory response (Ahmed et al., 2017), while a study in a similar model of optic nerve crush injury found that microglia, not peripheral monocytes, were the primary cells recruited to the site of injury (Heuss et al., 2018).

### A comparison of common microglial fate mapping systems

As diverse microglial fate mapping systems have proven useful for the analysis of microglial dynamics in development, homeostasis and disease, data has become available on the efficacies of various mouse strains in targeting microglia. Following the advent of *Cx3cr1^Cre^*-based models, their specificity for microglia was assessed and directly compared to earlier *Lyz2^Cre^* and *Cd11c^Cre^* mice, through direct crosses to *R26^YFP^* strains (Goldmann et al., 2013). While *Cx3cr1^Cre^*-targeted microglia with higher sensitivity and specificity than *Lyz2^Cre^* or *Cd11c^Cre^*, recombination was also observed in CX3CR1^+^ infiltrating peripheral myeloid cells. However, long-lived microglia, which retained a stable expression of YFP, could still be distinguished after a washout period of several weeks, while short-lived peripheral CX3CR1^+^ monocytes turned over rapidly and were replaced by YFP^–^ cells (Goldmann et al., 2013). Similar studies validated the utility of this approach as a means of distinguishing CX3CR1^+^ infiltrating monocytes and dendritic cells from resident parenchymal microglia (O’Koren et al., 2016; Mrdjen et al., 2018; Jordão et al., 2019), although it has been noted that some BAM subsets, such as those residing in the perivascular spaces meninges and CP, are replaced more slowly than other subsets and thus cannot be readily distinguished through this approach alone (Mrdjen et al., 2018; Jordão et al., 2019). Later work also established that many BAM subsets, in fact, express CX3CR1 (Goldmann et al., 2016). Accordingly, off-target recombination in non-parenchymal CNS macrophages is observed in *CX3CR^CreER^* systems (Chappell-Maor et al., 2020; Faust et al., 2023).

Recent advances in single-cell transcriptomics have identified a battery of novel microglia-enriched genes, several of which have been leveraged to generate Cre-based tools with applications to fate mapping. Examples include *Sall1* (Buttgereit et al., 2016), *Tmem119* (Kaiser and Feng, 2019), *P2ry12* (McKinsey et al., 2020), and *Hexb* (Masuda et al., 2020). *Sall1^CreER^* targets microglia with high sensitivity and specificity, notably showing no recombination in CNS-infiltrating peripheral myeloid cells and nearly all non-parenchymal BAMs with the exception of a small subset of CP BAMs (Buttgereit et al., 2016; Van Hove et al., 2019; Chappell-Maor et al., 2020). However, *Sall1* is a critical regulator of microglial homeostasis (Buttgereit et al., 2016), likely rendering this approach less suitable for microglial fate mapping in pathological states. Furthermore, off-target recombination was recently reported in neuroectoderm-derived CNS cells in *Sall1^CreER^* mice (Chappell-Maor et al., 2020). Likewise, the *Tmem119*^*CreERT*2^ line (Kaiser and Feng, 2019) labeled adult microglia with high specificity, although some recombination was noted in leptomeningeal macrophages and other non-IBA1^+^ cells thought to be perivascular or meningeal fibroblasts (Kaiser and Feng, 2019; McKinsey et al., 2020). The *P2ry12^CreER^* mouse line was developed with the goal of preserving endogenous expression of the functional P2RY12 receptor, which plays important roles in nucleotide-sensing during the microglial response to injury, seen as a benefit in contrast to the reduced expression of *Sall1* in *Sall1^CreER^* knock-in strains (McKinsey et al., 2020). The *P2ry12^CreER^* specifically and efficiently labeled embryonic as well as adult microglia, offering an advantage over the *Tmem119*^*CreERT*2^ construct, which mediated off-target recombination in CD31^+^ endothelial cells during development (Kaiser and Feng, 2019; McKinsey et al., 2020). No off-target recombination was observed in perivascular macrophages or in circulating blood cells, although a subset of CP macrophages were labeled, possibly corresponding to the microglia-like Kolmer’s epiplexus BAMs described by Van Hove et al. (2019) and McKinsey et al. (2020). However, like *Sall1*, *Tmem119*, and *P2RY12* are strongly associated with homeostatic microglial signatures (Kaiser and Feng, 2019; McKinsey et al., 2020). In fact, it was proposed that *Tmem119*-based systems could potentially be studied as sensors of disease states, as the expression of this gene may be perturbed under pathological conditions (Ruan et al., 2020). *Hexb*-based genetic tools have also recently been developed (Masuda et al., 2020). Massively parallel single-cell RNA-sequencing identified *Hexb* as a highly enriched gene in microglia that, unlike *Tmem119*, *Sall1*, or *P2ry12*, is consistently expressed across conditions of homeostasis and disease. *Hexb*^*CreERT*2^*:R26^YFP^* mice showed highly specific recombination in microglia, with distinct advantages over *Cx3cr1*- and *Sall1*-based systems (Masuda et al., 2020). However, although fate mapping validation experiments showed that *Hexb*^*CreERT*2^ did not label other CNS macrophage subsets or infiltrating peripheral monocytes, a measurable subset of microglia were not labeled (Masuda et al., 2020). Additionally, a recent study demonstrated that *Tmem119^CreER^* and *Hexb^CreER^* lines show low recombination efficiency relative to the established *Cx3cr1^CreER^* based tools (Faust et al., 2023). Recombination efficiency was shown to depend on the genetic distance separating the two loxP sites, with shorter inter-loxP distances promoting efficient recombination (Faust et al., 2023). Thus, further optimization of *Tmem119*-, *Sall1*-, *Hexb*-, *P2ry12*-, and *Cx3cr1-*based tools using evidence-driven methods will likely be required to exploit their full potential.

The previously described *CX3CR1^CreER^:R26^Confetti^* model offers a unique advantage in that fate mapping can be achieved with single-cell resolution (Tay et al., 2017). This has allowed detailed analyses of microglial dynamics under homeostatic and pathological conditions, greatly enhancing our ability to examine heterogeneity within the microglial population and track the migration and proliferation of individual cells (Tay et al., 2017). In the context of a recent explosion of microglial single-cell transcriptomics studies, approaches of this nature hold great promise as key techniques in the study of microglia at an unprecedented resolution.

## Translational applications

**FIGURE 4:**
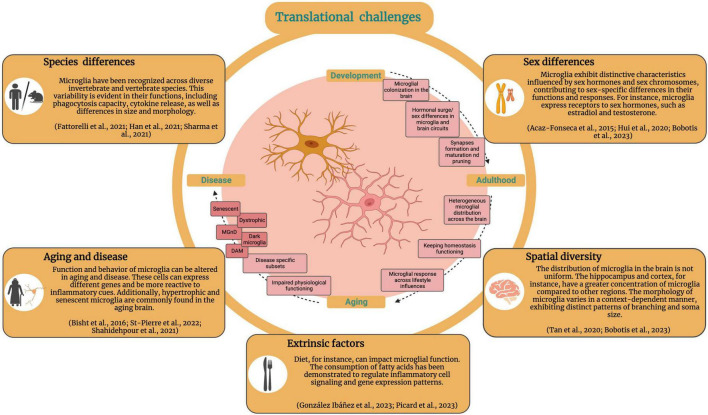
Translational applications for the study and targeting of human microglia. Current methods and associated challenges for the study of human microglia and translation of microglia-targeted therapies to the clinic. Created with BioRender.com.

In contrast to animal models, visualization of microglia in the living human brain remains a significant challenge. There are, however, several emerging techniques (Janssen et al., 2018), which can enable the study of microglial dynamics in living humans (Figure 4). A powerful technique used in clinical and fundamental research to study brain immunity is positron emission tomography (PET) (Gerhard et al., 2003; Di Biase et al., 2017; Hu et al., 2020). PET utilizes the binding of radioactive isotopes to a target molecule to visualize and quantify that molecule (Berger, 2003). PET has already highlighted the activity of microglia in contexts of neurodegeneration, inflammation and neurodevelopmental disorders, as measured using the translocator protein (TSPO) as a target (Yankam Njiwa et al., 2017; Hu et al., 2020; Lavisse et al., 2021; Seelaar and Van Swieten, 2021; Simpson et al., 2022; Vainio et al., 2022). Furthermore, microglia were shown to be highly active in people with schizophrenia using TSPO, as the upregulation of TSPO correlates with higher mitochondrial activity (Conen et al., 2021). However, TSPO is not specific to microglia, having been identified on astrocytes, endothelial cells and neurons and has been shown to vary considerably among brain regions (Daugherty et al., 2013; Betlazar et al., 2018; Gong et al., 2019). TSPO also demonstrates greater selectivity for microglia and distinct expression kinetics during inflammation in rodents compared to humans (Nutma et al., 2021a,b), limiting the translational potential of TSPO-based findings to the clinic. To address these issues, new ligands have been developed to target microglial proteins already common in animal research such as P2RY12, TREM1 and CSF1R, increasing the correlative research potential of the technique (Berdyyeva et al., 2019; Chaney et al., 2019, 2020; Zhou et al., 2021; Coughlin et al., 2022).

Diffusion-weighted magnetic resonance imaging (DW-MRI), which leverages the random motion of water molecules to obtain high-resolution microimaging, is emerging as a promising alternative strategy to PET for microglial study *in vivo*. DW-MRI has already been shown to detect changes in microglial proliferation, density morphology in living mice and rats (Yi et al., 2019; Garcia-Hernandez et al., 2022). Thus, DW-MRI could feasibly be applied as a non-invasive approach for the clinical study of microglia in living humans and remains an exciting field of research.

Methods to study human microglia *ex vivo* have also gained traction in recent years, with brain tissue samples obtained perioperatively from living donors yielding novel insights into human microglial phenotypes (Milior et al., 2019). In a sample of nine patients undergoing neurosurgery for refractory epilepsy, Morin-Brureau et al. (2018) provided a detailed characterization of the brain region-specific diversity of microglial morphologies and phenotypic marker expression. *Ex vivo* two-photon live imaging studies can also yield insights into human microglial dynamics, and have aided in elucidating P2RY12/13-dependent responses to ADP stimuli or tissue damage (Morin-Brureau et al., 2018; Milior et al., 2020). This cohort also provided a unique opportunity to study the correlation between seizure timing and frequency with microglial phenotype, with inferential data suggesting transient proinflammatory cytokine release in the microglial response to seizure (Morin-Brureau et al., 2018). Although access to brain tissue samples from living humans remains a challenge, the rapidly advancing study of *ex vivo* microglial dynamics promises to clarify roles for microglia in human pathologies, elucidating therapeutically relevant differences between microglial function in humans and animal models.

Finally novel *in vitro* models can assist in the study of human microglia, such as the differentiation of microglia from human induced pluripotent stem cells (Cuní-López et al., 2022). This additional *in vitro* model can be useful for recapitulating the features of authentic human microglia and enabling the cells to mature under CNS-derived conditions (Cuní-López et al., 2022). Plenty of protocols are emerging (Rustenhoven et al., 2016; Speicher et al., 2019), from primary to pluripotent stem cell-derived culture, and early results are beginning to elucidate a diversity of new mechanisms of human microglial function (Haenseler et al., 2017; Bassil et al., 2021; Popova et al., 2021; Dräger et al., 2022; Banerjee et al., 2023; Dolan et al., 2023).

## Discussion

Overall, research has progressed greatly since the initial description of microglia using silver staining in brightfield microscopy (Sierra et al., 2019). Significant advances in the methods used to study microglia have made it possible to characterize in detail the microglial sensome, define microglial subsets and their distinct phenotypic markers and elucidate the functional roles of diverse microglial genes and proteins in health and disease.

Antibody-mediated identification of microglia has evolved substantially as the diversity and complexity of these cells has been increasingly recognized. A key challenge to overcome has been to define the specificity of protein markers for microglia relative to other CNS-associated macrophages and non-immune cells, as well as their context-dependence and regulation during inflammation and pathology. The combination of multiple techniques, such as high-dimensional flow cytometry, FACS, and IHC has enabled increasingly detailed phenotyping of microglia, and our understanding of the sensitivity and specificity of microglial markers continues to grow. The advent of single-cell transcriptomics has enabled increasingly granular analysis of microglial phenotypes, with a particularly high volume of data in the contexts of development and neurodegeneration (Keren-Shaul et al., 2017; Krasemann et al., 2017; Hammond et al., 2019; Li et al., 2019a; Olah et al., 2020), and is already yielding valuable tools for the antibody-mediated identification of specific functional subpopulations as well as the broad visualization of microglia across diverse spatial and temporal settings.

In parallel, sophisticated modern imaging techniques have granted researchers the capacity to image microglial dynamics at a single-cell resolution through two-photon *in vivo* imaging and gain unprecedented insights into microglial ultrastructure and their interactions with neighboring cells and the extracellular environment through EM (Maco et al., 2014; Nahirney and Tremblay, 2021). These techniques will continue to enhance our understanding of the relationship between microglial structure and function, complementing the large body of work leveraging microglial markers to study functional properties.

Selective microglial depletion is becoming increasingly accessible as our understanding of the roles and specificity of microglial genes and proteins continues to grow. These studies have shed light on the critical functions of microglia as a whole, as well as their individual components, across an expanding list of disease settings. Studies investigating the highly specific genetic or pharmacological depletion of increasingly relevant microglial phenotypes promises to advance microglia-targeted therapies for currently untreatable diseases, as exemplified by recent elucidations of the roles of *Trem2*, *Apoe*, *Clec7a* and others in neuropathological conditions (Wang et al., 2015, 2022; Krasemann et al., 2017; Deerhake et al., 2021).

Gene reporter and fate-mapping systems have gradually improved as models to investigate microglial development, homeostatic function and roles in pathology. Cre-based systems have often been limited by sparse knowledge of microglial and myeloid gene expression patterns and inherent flaws such as spontaneous recombination or low recombination efficiency (Chappell-Maor et al., 2020; Van Hove et al., 2020; Faust et al., 2023). However, as our understanding of microglial markers has improved, so too has our ability to exploit these markers to selectively label, visualize and track microglia. As recently demonstrated, the application of single-cell RNA-sequencing could be a promising approach to identify future genetic targets with high specificity to microglia (Masuda et al., 2020). As more options for microglial reporter systems continue to emerge, systematic comparisons of the efficiency, selectivity and generalizability of these models have been and will be critical in aiding researchers to confidently select the most appropriate reporter model for their application (Faust et al., 2023). With recent advances enabling the visualization of individual microglial cells (Tay et al., 2017) and selectively studying microglia with high specificity (Kim J.-S. et al., 2021), this area of research holds high potential to answer some of the most essential questions in microglial biology.

The translation of microglia-targeted therapies remains to the clinic remains limited to date, and characterizing microglial phenotypes and functions in humans will be critical to achieve this. Species-specific differences may greatly impact the translation of microglia-targeted therapies (Fattorelli et al., 2021), and more work is needed to understand human microglial gene expression and function in order to modulate these for therapeutic purposes. Consideration must also be given to factors such as sex in the context of clinical translation, as microglial density, size, phagocytic activity, morphology and target marker expression can vary between sexes (Bobotis B. C. et al., 2023), and this is also species-dependent (Han et al., 2021; Sharma et al., 2021). For instance, sexual dimorphism is observed in diseases such as AD, when assessing post-mortem brain tissue samples, female patient microglia exhibited diverse morphologies in contrast to male samples, predominantly amoeboid with increased CD68 immunoreactivity (Guillot-Sestier et al., 2021). Moreover, microglia are also influenced by disease-specific context, aging, brain location and extrinsic factors such as medications and drugs (Savchenko et al., 1997; Bollinger et al., 2017; Wang et al., 2021; VanderZwaag et al., 2023), presenting a significant obstacle to the translation of microglia-targeted therapies.

With the core proteins and genes discussed in this review, as well as established and emerging techniques for investigating microglia in homeostasis and disease, our knowledge of microglia and their roles in pathology is continuing to grow exponentially. Given the spatial complexity and heterogeneity of microglia in terms of spatial and temporal characteristics (Carrier et al., 2020), it is critical to employ a broad variety of complementary techniques in integration in order to fully characterize all aspects of this unique subset of immune cells. As microglia are increasingly implicated in a growing list of CNS diseases, optimizing these tools to improve the study of microglia promises to enhance our understanding of fundamental concepts in microglial biology and elucidate novel therapeutic targets with important clinical implications.

## Author contributions

BB: Conceptualization, Project administration, Writing – original draft, Writing – review & editing. TH: Conceptualization, Writing – original draft, Writing – review & editing. MC: Writing – original draft, Writing – review & editing. M-ÈT: Funding acquisition, Resources, Supervision, Writing – original draft, Writing – review & editing.
